# HER2 and EGFR amplification and expression in urothelial carcinoma occurs in distinct biological and molecular contexts

**DOI:** 10.18632/oncotarget.16554

**Published:** 2017-03-24

**Authors:** Pontus Eriksson, Gottfrid Sjödahl, Gunilla Chebil, Fredrik Liedberg, Mattias Höglund

**Affiliations:** ^1^ Division of Oncology and Pathology, Department of Clinical Sciences, Lund University, Lund, Sweden; ^2^ Division of Urological Research, Department of Translational Medicine, Lund University, Skåne University Hospital, Malmö, Sweden; ^3^ Unilabs, Helsingborg Hospital, Helsingborg, Sweden

**Keywords:** HER2, EGFR, amplification, urothelial carcinoma, molecular subtype

## Abstract

We analyzed a cohort of 599 cases of urothelial carcinoma for *EGFR*, *ERBB2*, and *ERBB3* gene expression and genomic alterations. The cohort consisted of a reference set (*n* = 292) comprising all stages and grades and one set (*n* = 307) of advanced tumors. All cases were previously classified into urothelial carcinoma molecular subtypes. Genomic amplifications were established by array-CGH or *in-situ* hybridization, and gene expression both at mRNA and protein levels. Clinical HER2 status was independently evaluated using standard clinical procedures. *EGFR* amplifications were observed in 14% and *ERBB2* amplifications in 23% of the reference cohort. *EGFR* gains were enriched in the Basal/SCC-like and *ERBB2* gains in the Genomically Unstable subtypes. The expression data suggests that the Genomically Unstable show high *ERBB2/ERBB3* but low *EGFR* expression and that Basal/SCC-like tumors show high *EGFR* but low *ERBB2/ERBB3* expression. Whereas the frequency of ERBB2 genomic amplification were similar for cases of the Genomically Unstable subtype in the two cohorts, the Urothelial-like subtype acquires *ERBB2* amplifications and expression during progression. Even though a good correlation between gene amplification and ERBB2 gene expression was observed in the Urothelial-like and Genomically Unstable subtypes less than half of the Basal/SCC-like cases with *ERBB2* amplification showed concomitant *ERBB2* mRNA and protein expression. We conclude that clinical trials using ERBB2 (HER2) or EGFR as targets have not fully appreciated the molecular heterogeneity in which activated ERBB2 and EGFR systems operate. Proper tumor classification is likely to be critical for arriving at thorough conclusions regarding new HER2 and EGFR based treatment regimes.

## INTRODUCTION

Bladder cancer is the fifth most common malignancy in the Western world. It is associated with a high rate of mortality in patients with advanced disease. In spite of this, few significant improvements in survival has been achieved during the last three decades [1]. While a proportion of patients respond to conventional chemotherapy these responses are rarely long-lasting. Even though molecular targeted therapy has been successful for other cancers types, few advances have been made in bladder cancer. In analogy with other tumor types, members of the ERBB receptor family have been considered as potential targets also for urothelial carcinomas *e.g*., ERBB2 (HER2) [2] and EGFR [3]. In urothelial carcinomas, overexpression of ERBB2 protein has been reported to vary considerably between studies [4] as well as between geographically distinct cohorts [5]. Several studies have also reported a low concordance between ERBB2 protein level and gene amplification in urothelial carcinomas [5, 6]. Hansel *et al*. detected ERBB2 overexpression (IHC) in 36% but genomic amplification in only 10% of tumors [7]. Similarly low levels of *ERBB2* genomic amplifications have also been reported by other investigators (*e.g*., [8]). Hence no consensus regarding the status of HER2 alterations in urothelial cancer has been arrived at. Furthermore, even though several trials aiming for HER2 as a target in bladder cancer have been initiated, no general conclusions have been reached. A factor contributing to the disparate results is most likely the underlying heterogeneity of bladder cancer and the lack of adequate molecular descriptions of bladder cancer molecular subtypes; the context in which the ERBB2 and EGFR targets operate. We have described three major molecular subtypes of bladder cancer, Urothelial-like (Uro) (previously termed Urobasal [9]), Genomically Unstable (GU) and Basal/SCC-like [9], that accommodates fundamental differences in their molecular biology [10–14]. By combining global mRNA gene expression and extensive immunohistochemistry (IHC) analyses we have shown that the Uro, GU, and SCC-like subtypes dominate also the muscle invasive tumors, albeit in more progressed and infiltrated versions [15]. Here we investigate *EGFR*, *ERBB2* (HER2), and *ERBB3* (HER3) genomic alterations and expression in relation urothelial carcinoma molecular subtypes. To be able to study changes during tumor progression we analyze two cohorts, one (*n* = 292) dominated by non-muscle invasive tumors and a second (*n* = 307) dominated by muscle invasive tumors. We show that *ERBB2* amplifications and expression, as well as clinically “HER2 positive” cases, may be of two fundamentally different molecular subtypes, of Uro or the GU subtypes, and that EGFR expression is associated with the SCC-like subtype.

## RESULTS

### EGFR, ERBB2, and ERBB3 alterations in the reference cohort

**Figure 1:**
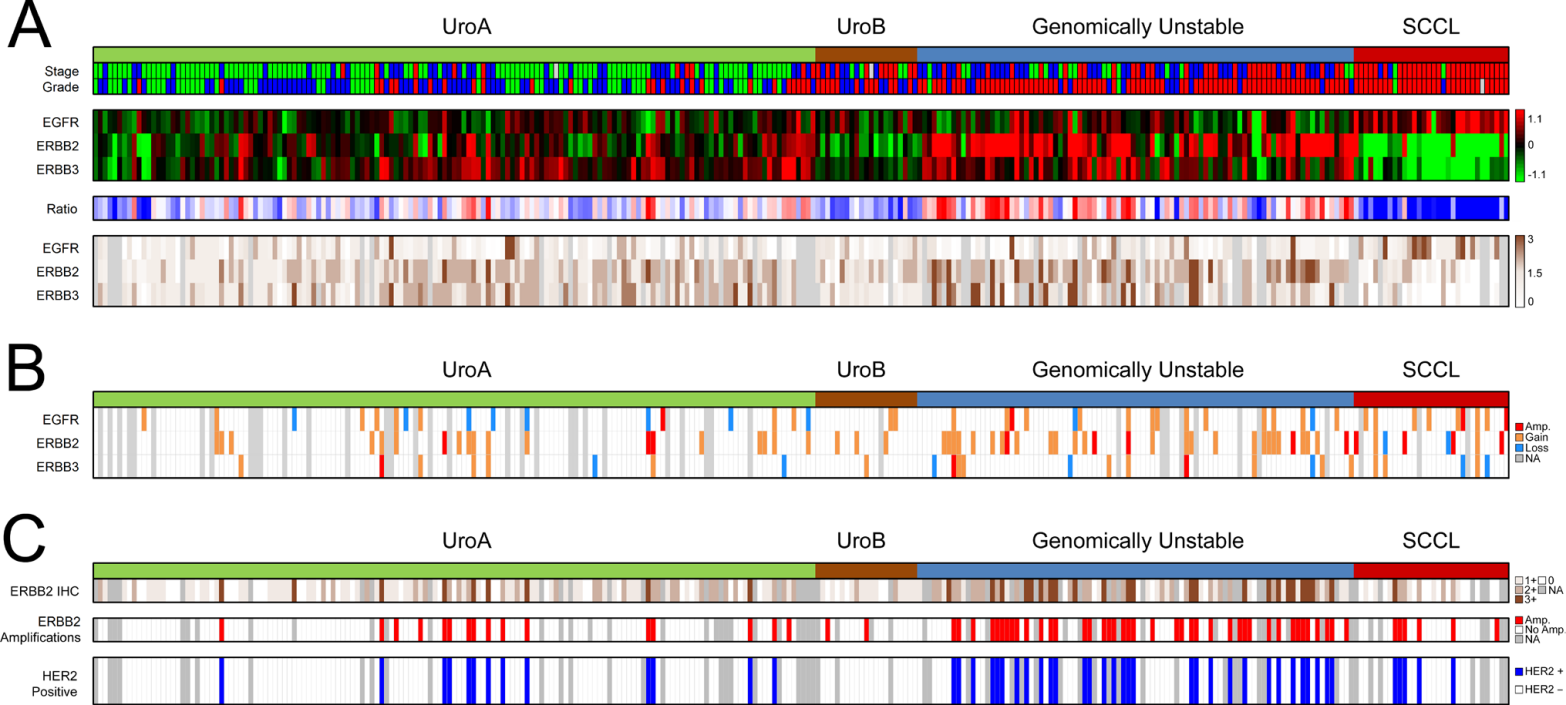
Summary of gene expression, IHC scoring, copy number alterations, and Clinical HER2 status in the reference cohort (**A**) Panel 1: Molecular subtypes and tumor stage and grade. Stage: Ta, green; T1, blue; MI, red. Grade: G1, green; G2, blue; G3, red. White indicates missing information. Panel 2: Gene expression of *EGFR, ERBB2*, and *ERBB3* for 292 samples ordered by subtype. Red, high expression; green, low expression. Panel 3: The ratio of *EGFR*/ *ERBB2* and *ERBB3* mRNA expression emphasizes the subtype specific pattern of expression. Red, high ERBB2 and ERBB3 expression, and low EGFR expression; blue, low ERBB2 and ERBB3 expression, and high EGFR expression. Panel 4: EGFR, ERBB2, and ERBB3 immunohistochemistry scores. The score represents a tumor cell score (TCS), where the staining intensity is multiplied by the fraction of the tumor cells that show staining. Dark brown, high expression; white, no expression; gray, missing data. (**B**) Copy number alterations as determined by array-CGH. Red, focal amplification; orange, gain; blue, loss; gray, missing data. (**C**) Clinical HER2-status evaluated using clinical guidelines. Panel 1: ERBB2 (HER2) IHC intensity scores (0–3) in > 10% of tumor cells. White, score = 0; light brown, score = 1, brown, score = 2; dark brown, score = 3; gray, missing data. Panel 2: HER2 amplifications as determined by *in-situ* hybridization (ISH) (*ERBB2*/CEN17 ISH ratio ≥ 2 or ≥ 4 *ERBB2* copies). Red, ERBB2 amplification; white, no amplification; gray, missing data. Panel 3: HER2-positive samples (IHC2+ with HER2 amplification). Blue, HER2 positive; white, HER2 negative; gray, missing data. All panels show the 292 samples.

**Figure 2:**
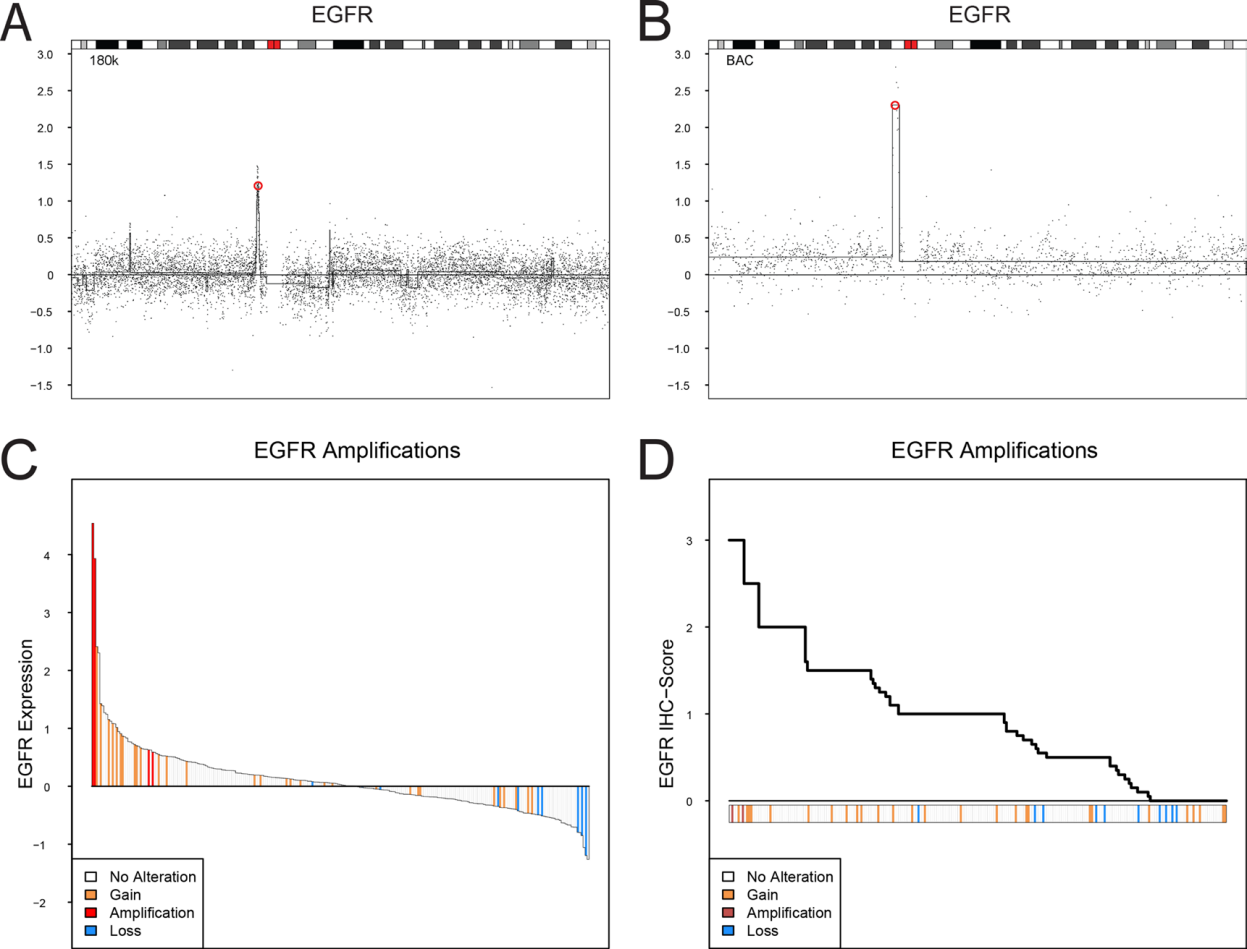
EGFR copy number alterations, mRNA expression, and protein expression (IHC) (**A** and **B**). Examples of focal amplification events spanning *EGFR* on chromosome 7. The genomic position of *EGFR* is indicated by a red circle. (**C**) Ranked mRNA gene expression levels for 249 samples with both gene expression and copy number aberration data. (**D**) Ranked EGFR immunohistochemistry scores (TCS) for 251 samples with both IHC and copy number aberration data. EGFR gene copy number levels: focal amplification, red; gain, orange; loss, blue.

**Figure 3:**
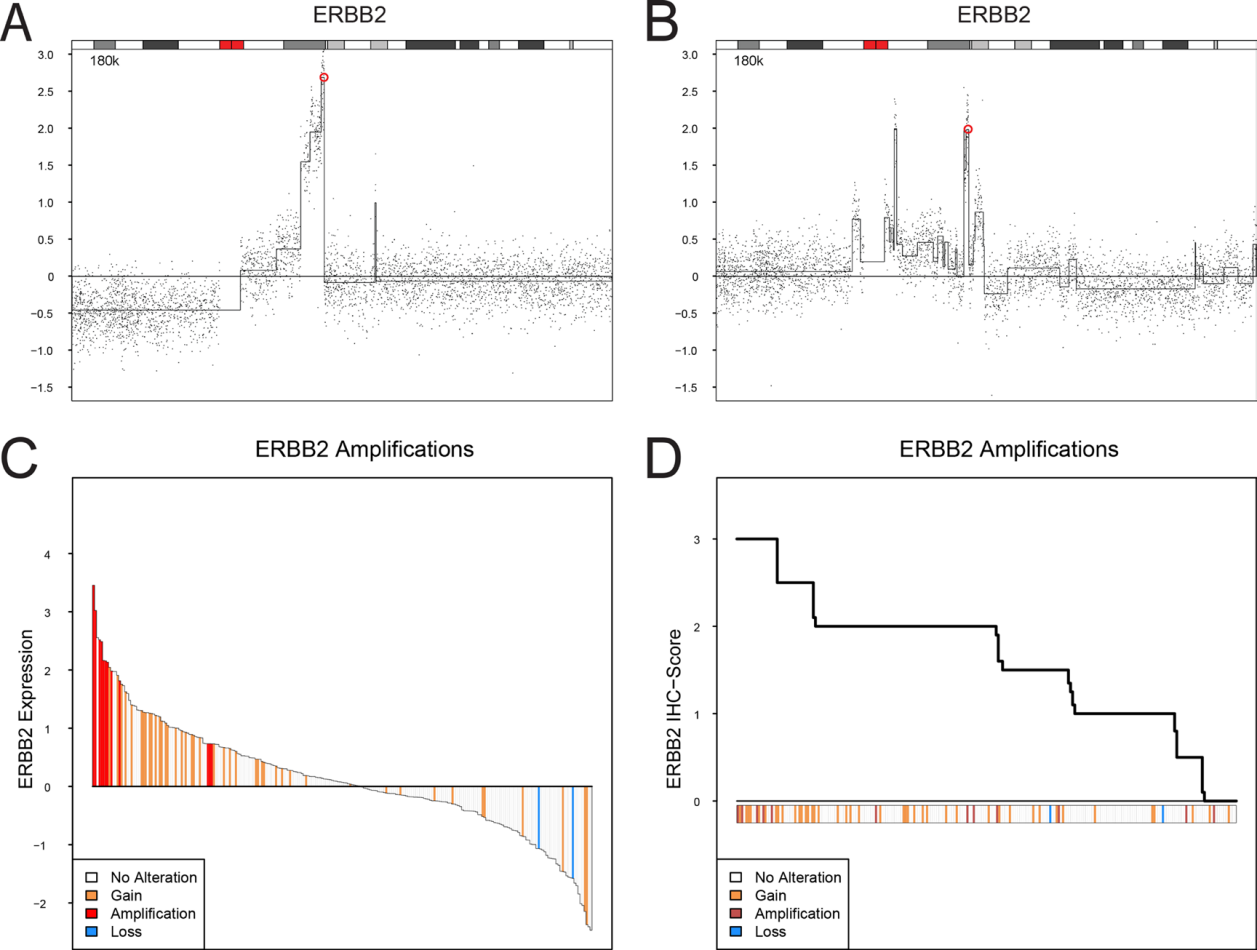
ERBB2 (HER2) copy number alterations, mRNA expression, and protein expression (IHC) (**A** and **B**). Examples of focal amplification events spanning *ERBB2* on chromosome 17. The genomic position of *ERBB2* is indicated by a red circle. (**C**) Ranked mRNA gene expression levels for 249 samples with both gene expression and copy number aberration data. (**D**) Ranked ERBB2 (HER2) immunohistochemistry scores (TCS) for 251 samples with both IHC and copy number aberration data. ERBB2 gene copy number levels: focal amplification, red; gain, orange; loss, blue.

In this investigation we used two cohorts, one reference cohort consisting of 292 cases, and one cohort of 307 advanced cases. The reference cohort was established from previously published data [10, 12] and included all tumor stages (123 Ta, 88 T1, 79 ≥T2, and 2 Tx) and grades (54 G1, 96 G2, 141 G3, and 1 Gx, WHO1999). Tumors were subtype classified as Urothelial-like A (UroA, *n* = 149), Urothelial-like B (UroB, *n* = 21), Genomically Unstable (GU, *n* = 90), or Squamous Cell Carcinoma-like (SCC-like, *n* = 32) [10]. In the reference cohort we observed significantly increased *EGFR* mRNA (*p* = 1.5 × 10^−4^) and protein expression (*p* = 10^−6^) in the SCC-like subtype, whereas *ERBB2* mRNA and protein expression levels were significantly higher in the GU subtype (*p* = 10^−13^, and *p* = 10^−8^, respectively) (Figure 1A). The *ERBB3* mRNA and protein levels were higher within the UroA and GU tumors, and significantly correlated with ERBB2 expression both at the mRNA (*r* = 0.52, *p* = 10^−15^) and protein levels (*r* = 0.59, *p* = 10^−15^). The data suggests that particularly GU tumors show high *ERBB2*/*ERBB3* expression and low *EGFR* expression, and SCC-like tumors the opposite pattern (Figure 1A). The copy number array data indicated *EGFR* (7p11) gains in 30 tumors (12%), and focal genomic amplifications in another 4 cases (1.6%) (Figure 1B, Figure 2A and 2B). Even though *EGFR* gains and amplifications occurred more often in both GU and SCC-like subtypes, only the latter showed significant enrichment (*p* = 0.008, Fisher's exact test). *EGFR* gains and amplifications influenced both mRNA levels (*p* = 10^−13^) and protein expression (*p* = 2 × 10^−4^) significantly (Figure 2C and 2D). *ERBB2* (17q12) showed copy number gains in 45 (18%) and focal genomic amplifications in 12 samples (5%) (Figure 1B, Figure 3A and 3B). Gains were significantly more common in the GU subtype (*p* = 0.001). Gains and focal amplifications affected both mRNA and protein levels significantly (*p* = 10^−16^ and *p* = 10^−5^, respectively) (Figure 3C and 3D), however, only 34% of the tumors with ERBB2 IHC TCS staining ≥ 2 (Tumor Cell Score, See Materials and Methods) had *ERBB2* copy number gains detectable with array-CGH (Figure 3D). Overall, gains or amplifications of *EGFR* and *ERBB2* were seen in 14% and 23% across UC molecular subtypes, respectively. *ERBB3* (12q13) copy number alterations were scarce, and not significantly associated with mRNA or protein expression (Figure 1A and 1B, Supplementary Figure 1).

Co-occurring focal amplifications of *EGFR*, and *ERBB2* or *ERBB3* were not observed, while co-occurring gains of *EGFR* and *ERBB2*, and *EGFR* and *ERBB3* were seen in eight and one cases, respectively. Five cases showed gains of all three genes. Examining the very high expressing cases (with Tumor Cell Score ≥ 2.5, indicating high protein expression throughout the tumor), EGFR+ERBB2 expression was only observed in one GU tumor, while two UroA tumors expressed both EGFR and ERBB3 at high levels. None of the tumors had strong simultaneous expression of all three proteins. Very high simultaneous expression of both ERBB2 and ERBB3 was seen in 11 tumors, where 6 were GU and 5 were UroA.

In a clinical setting, HER2 positivity is established by both HER2 expression (IHC) and amplification status (dual probe *in-situ* hybridization). Of the original cohort of 292 cases, 232 could be evaluated by both methods. A tumor was considered HER2 positive if IHC 2+ staining was observed in ≥ 10% of tumor cells and ERBB2 was amplified (*ERBB2*/CEN17 ratio ≥ 2 or nERBB2 ≥ 4). In total, 49 out of 232 (21%) tumors were considered HER2 positive (Figure 1C), with a strong enrichment in the GU subtype with 31 out of 69 (45%) cases considered HER2 positive (*p* = 4 × 10^−8^). Among the tumors with IHC 2+ scores, 38% (22/58) were considered amplified by *in-situ* hybridization, whereas 82% (27/33) of the cases with IHC 3+ scores were considered amplified.

### *EGFR*, *ERBB2* and *ERBB3* in advanced urothelial carcinoma cohort

**Figure 4:**
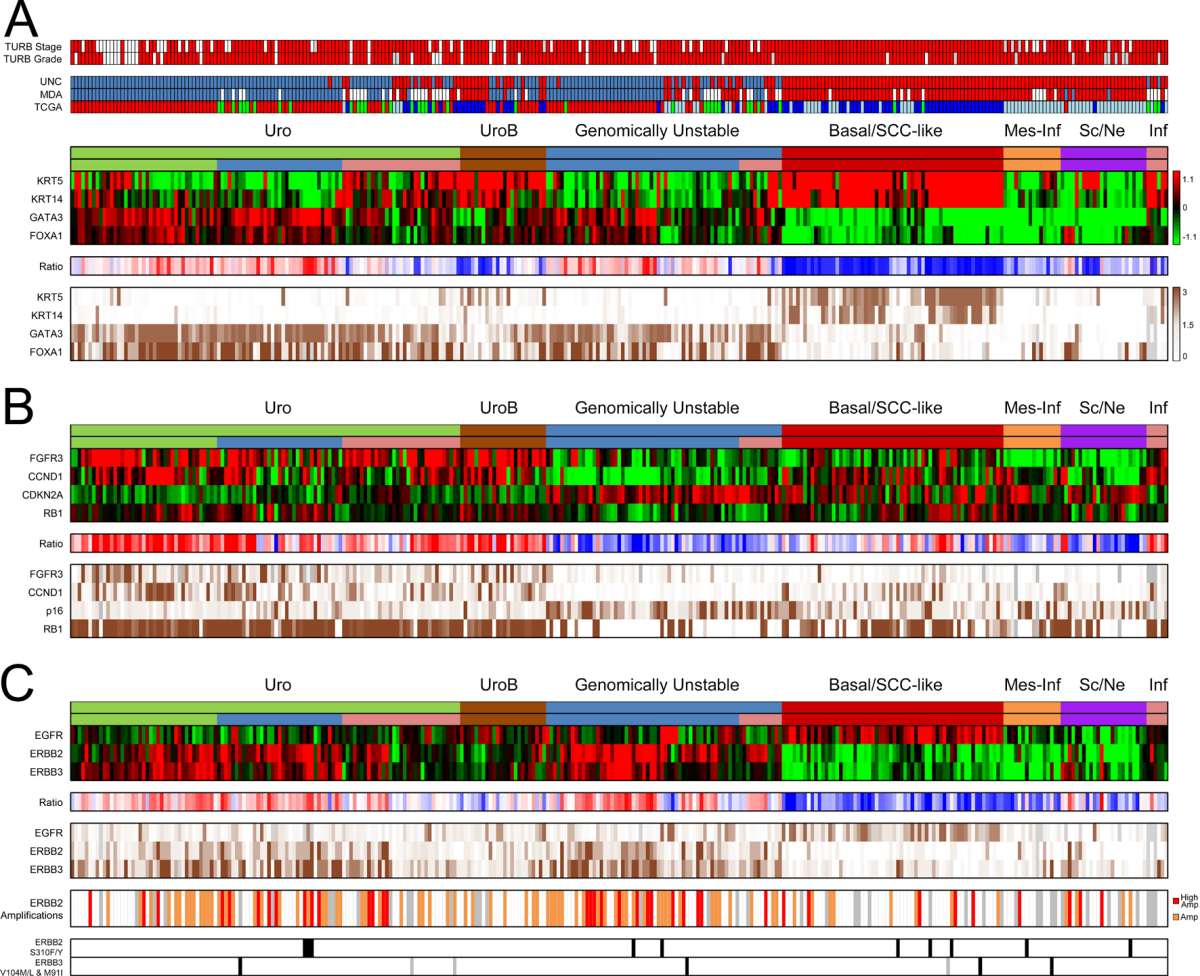
Summary of gene expression, IHC scoring, and copy number alterations in the advanced UC cohort (**A**) Panel 1: TURB pathological stage and grade. Stage ≥ T2, red; < T2, white. Grade G3, red; < G3, white; gray, missing data. Panel 2: For comparison; nearest centroid classification of the cohort using centroids derived from the UNC, MDA, and TCGA classification systems, respectively. UNC classification, color code; blue, Luminal; red, Basal. MDA classification, color code; blue, Luminal; white, p53-like; red, Basal. TCGA classification, color code; red, Cluster I, green; Cluster II; blue, Cluster III; lightblue, Cluster IV. Panel 3: mRNA expression of consensus marker genes KRT5, KRT14, GATA3, and FOXA1 indicates the Basal/SCC-like phenotype. The top subtype header indicates the final subtyping derived from the combined interpretation of both mRNA and IHC data. Among the Uro tumors, the lower bar indicates the advanced UroA tumors in green, while the UroC group is indicated in blue. The pink groups in the Uro and GU subtype indicate tumors with high immune and stromal cell content. Panel 4: Ratio of *KRT5* and *KRT14*, and *GATA3* and *FOXA1* mRNA expression levels. Red, high FOXA1 and GATA3 expression, and low KRT5 and KRT14 expression; blue, low FOXA1 and GATA3 expression, and high KRT5 and KRT14 expression. Panel 5: Immunohistochemistry scores (TCS) of KRT5, KRT14, GATA3 and FOXA1. Dark brown, high expression; white, no expression; gray, missing data. (**B**) Panel 1: mRNA heat map of key genes *FGFR3*, *CCND1*, *CDKN2A*, and *RB1* that distinguish Uro from the GU subtype. Panel 2: Ratio between the mRNA levels of FGFR3, CCND1, and RB1, and CDKN2A (p16). Red, high FGFR3, CCND1, and RB1 expression, and low CDKN2A expression, blue, low FGFR3, CCND1, and RB1 expression, and high CDKN2A expression. Panel 3: Immunohistochemistry scores (TCS) of FGFR3, CCND1, CDKN2A (p16), and RB1. Dark brown, high expression; white, no expression; gray, missing data. (**C**) Panel 1: mRNA expression of *EGFR*, *ERBB2*, and *ERBB3*. Panel 2: Ratio between the mRNA expression of *ERBB2* + *ERBB3*, and *EGFR*. Red, high ERBB2 and ERBB3 expression, and low EGFR expression; blue, low ERBB2 and ERBB3 expression, and high EGFR expression. Panel 3: IHC scores (TCS) for EGFR, ERBB2, and ERBB3. Dark brown, high expression; white, no expression; gray, missing data. Panel 4: *ERBB2* amplifications. Orange, *ERBB2*/CEN17 ISH ratio ≥ 2 or ≥ 4 *ERBB2* copies; red, *ERBB2*/CEN17 ISH ratios ≥ 3 or ≥ 6 *ERBB2* copies. Panel 5: *ERBB2* S310F/Y mutations indicated in black, *ERBB3* V104M/L mutation indicated in black and M91I mutation in gray.

The 307 samples in the advanced cohort were all from patients that underwent radical cystectomy. The tumors in this cohort have been classified into molecular subtypes through the combination of global mRNA expression and extensive immunohistochemistry [15]. The combined analyses produced robust subtype assignments into Urothelial-like (Uro), Urothelial-like B (UroB), Genomically Unstable (GU), Basal/SCC-like, Mesenchymal Infiltrated (Mes-Inf), and Small Cell/Neuroendocrine (Sc/NE) (Figure 4). In addition, a small group (*n* = 6) of highly infiltrated, and thus unclassified, tumors were detected (Inf in Figure 4). The expression patterns defining the Basal/SCC-like subtype is illustrated in Figure 4A, with high KRT5 and KRT14, and low GATA3 and FOXA1 expression [9]. The majority of the non-Basal/SCC-like tumors belong to the Urothelial-like or Genomically Unstable category of tumors, which can be distinguished from each other by the expression patterns of FGFR3, CCND1, CDKN2A (p16), and RB1 (Figure 4B). The combination of mRNA and IHC data also enabled us to resolve the remaining tumor cell phenotypes where examination of mRNA expression patterns alone is complicated by infiltrating immune cells or high stromal cell content [15]. The final subtype distribution was 109 Uro (35.5%), 24 UroB (7.8%), 66 GU (21.5%), 62 Basal/SCC-like (20.2%), 16 Mes-Inf (5.2%), and 24 Small Cell/Neuroendocrine (Sc/NE) (7.8%) and 6 remaining Infiltrated (Inf) (2%).

Increased *EGFR* mRNA and protein levels were largely confined to the Basal/SCC-like subtype (*p* = 10^−12^ and *p* = 10^−13^, respectively) (Figure 4C). *ERBB2* mRNA expression was significantly higher within the Uro and GU subtypes (*p* = 10^−9^ and *p* = 10^−13^, respectively), an enrichment also seen at the protein level (*p* = 10^−7^ and *p* = 10^−10^). The *ERBB3* mRNA expression was highest in the Uro and GU tumors (*p* = 10^−16^ and *p* = 10^−9^), and was strongly correlated with the mRNA levels of *ERBB2* (*r* = 0.69, *p* = 10^−16^). The Uro and GU tumors also had the strongest ERBB3 protein expression. The EGFR, ERBB2, and ERBB3 protein expression showed a strong correlation with the mRNA levels (*r* = 0.65, 0.75, and 0.69, respectively, all *p* < 10^−16^) (Figure 4C). The ratio of *ERBB2*+*ERRB3* to *EGFR* mRNA expression summarizes the overall expression patterns and identifies Uro and GU tumors as ERBB2, ERBB3 high and EGFR low, and the Basal/SCC-like tumors as EGFR high and ERBB2, ERBB3 low (Figure 4C). No tumors had very high simultaneous EGFR and ERBB2 protein expression (IHC Tumor Cell Score ≥ 2.5), while one Uro tumor had high EGFR and ERBB3 protein expression. Similarly, no tumor had simultaneous high expression of all three proteins. There were 22 tumors with very strong ERBB2 and ERBB3 protein expression (9 Uro, 12 GU, and 1 Sc/Ne).

Copy number gain of *ERBB*2 was assessed using dual *in-situ* hybridization, with *ERBB2*/CEN17 ratios obtainable for 261 patients and an additional 22 with *ERBB2* counts only. In total, 116 tumors (37.7%) were deemed amplified (*ERBB2*/CEN17 ratio ≥ 2 or nERBB2 ≥ 4). Amplifications were seen in 45% of Uro (49/109), 29.2% of UroB (7/24), 59% of GU (39/66), 21% of Basal/SCC-like (13/62), 6.3% of Mes Inf (1/16), and 29% of Sc/NE (7/24) (Figure 4C). Out of the 116 amplifications 37 were considered high level amplifications, defined here as *ERBB2*/CEN17 ratio ≥ 3 or nERBB2 ≥ 6. Of these, 15 were of the Uro subtype, 13 GU, 5 Basal/SCCL, 1 Mes-Inf, and 3 of the Sc/Ne subtype. As other investigators, we observed a large proportion of cases (*n* = 44) with increased ERBB2 protein expression (IHC score ≥ 2+) without gene amplification. These cases were primarily of the Uro (*n* = 20) and the GU (*n* = 15) subtype. Amplifications in the Basal/SCC-like subtype differed from those in Uro and GU as less the half of these cases (6/13) showed strong ERBB2 protein expression (IHC score ≥ 2+). The overall amplification rate in IHC 2+ and 3+ scoring tumors were 50% and 90%, respectively. Hotspot mutation analysis of S310F/Y in ERBB2 and V104L/M and M91I in ERBB3 revealed low mutation frequencies and no subtype associations (Figure 4C).

**Figure 5:**
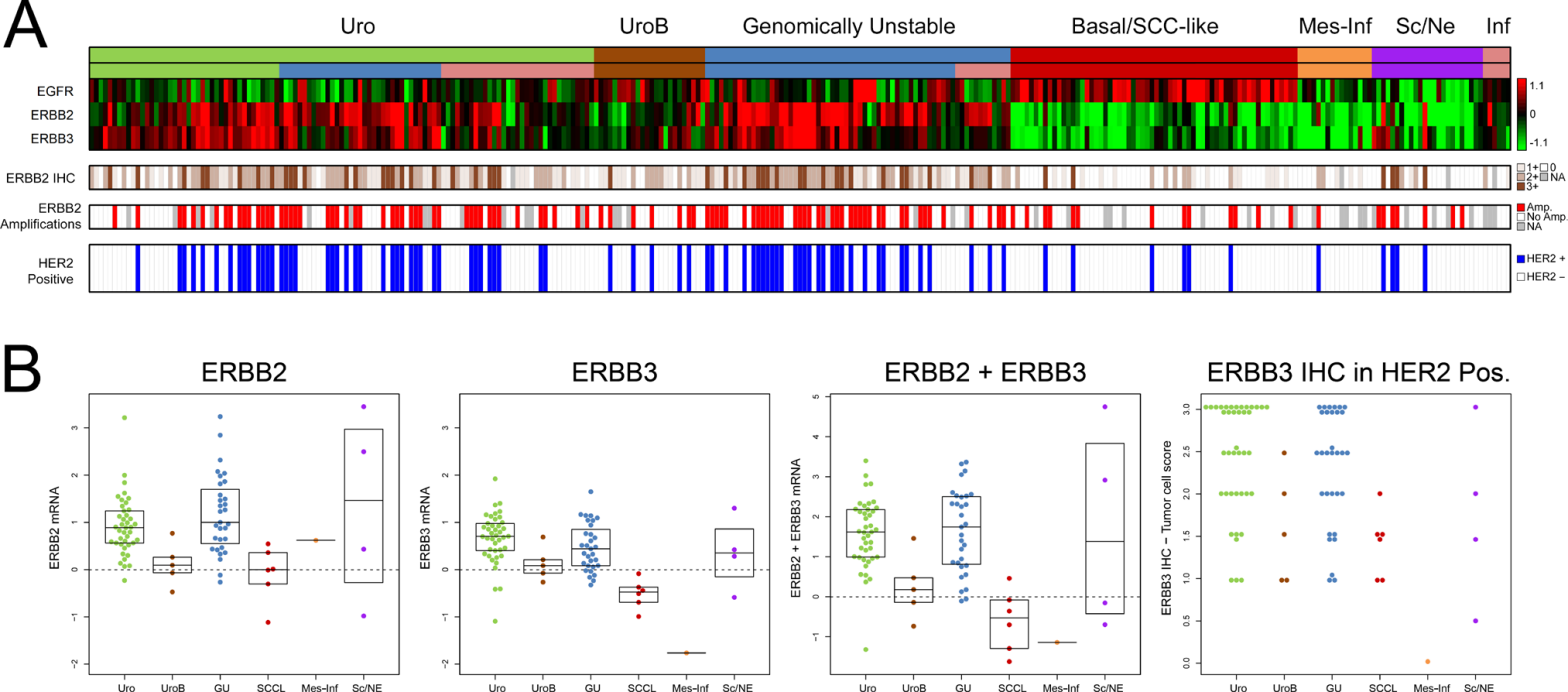
Clinical HER2 status in the advanced UC cohort (**A**) Panel 1: mRNA expression of *EGFR*, *ERBB2*, and *ERBB3*. Panel 2: ERBB2 (HER2) IHC intensity scores (0–3) in > 10% of tumor cells. White, score = 0; light brown, score = 1, brown, score = 2; dark brown, score = 3; gray, missing data. Panel 3: HER2 amplifications as determined by *in-situ* hybridization (*ERBB2*/CEN17 ISH ratio ≥ 2 or ≥ 4 *ERBB2* copies). Red, ERBB2 amplification; white, no amplification; gray, missing data. Panel 4: HER2-positive samples (IHC2 + with HER2 amplification). Blue, HER2 positive; white, HER2 negative; gray, missing data. (**B**) Boxplots illustrating subtype mRNA levels of *ERBB2*, *ERBB3*, and summed *ERBB2*+*ERBB3* expression in the HER2-positive tumors. The rightmost boxplot indicates the ERBB3 immunohistochemistry score in HER2 positive cases.

The advanced UC tumors were evaluated for clinical HER2 status (Figure 5A). In total, 88 tumors (29%) were deemed HER2 positive. The proportion of HER2 positive tumors was 38% (41/109) in Uro, 21% (5/24) in UroB, 47% (31/66) in GU, 10% (6/62) Basal/SCC-like, 6% (1/16) in Mes-Inf, 17% (4/24) in Sc/NE, and none in the remaining six infiltrated tumors. Notably, HER2 positive Uro and GU patients showed higher ERBB3 mRNA and protein expression compared to the other subtypes, indicating a different context of ERBB2 amplification and overexpression (Figure 5B). The Uro tumors may be divided into one group that contain the majority of the < T2 and < G3 tumors found in the dataset (indicated by green in the lower subtype bar in Figure 5), and in a more progressed UroC group (indicated by blue in the lower subtype bar in Figure 5) defined in Sjödahl *et al*. 2017 [15] with almost exclusively ≥ T2 G3 tumors. The latter group represents a progressed form of Uro distinct from UroB. The advanced Uro tumors exhibited a 34% (14/41) HER2 positive frequency while the UroC group showed a frequency of 54% (19/35), in stark contrast to the 11% seen in non-muscle invasive UroA tumors in the reference cohort (Figure 1). In contrast, GU tumors were HER2 positive in 45% the reference cohort and in 47% in the advanced cohort.

## DISCUSSION

**Figure 6:**
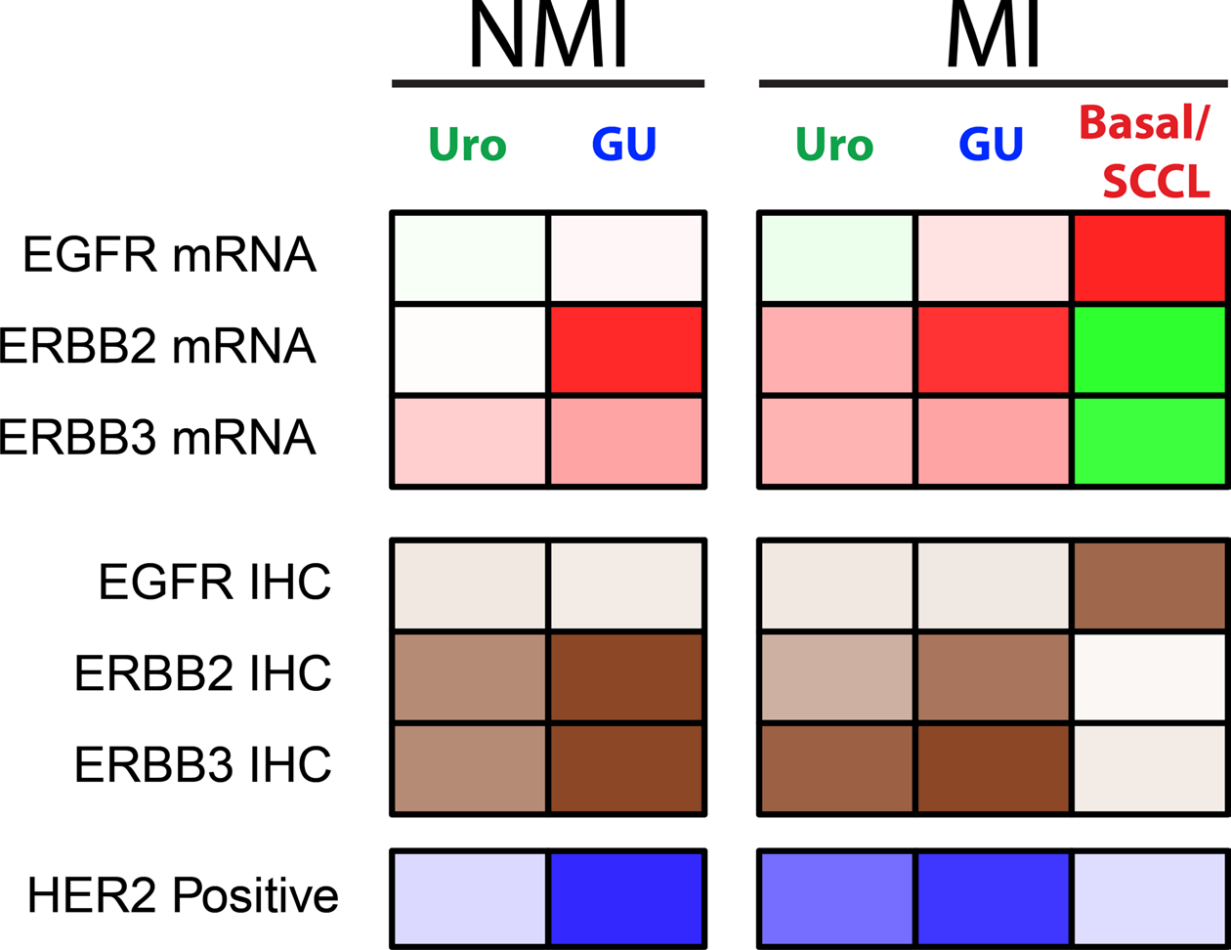
Summary of results in relation to molecular subtypes Uro NMI, includes UroA and UroB Ta or T1 tumors; GU NMI, includes GU Ta and T1 tumors; Uro MI, includes Uro, UroB, and UroC cases from the series of advanced tumors; GU MI, includes GU cases from the series of advanced tumors; Basal/SCCL MI, includes Basal/SCC-like cases from the series of advanced tumors. Non muscle invasive cases of Basal/SCC-like were too few to be included. Panel 1: Summary of EGFR, ERBB2, and ERBB3 mRNA expression. Red, high expression; green, low expression. Panel 2: Summary of EGFR, ERBB2, and ERBB3 protein expression. Dark brown, high protein expression; white, low protein expression. Panel 3: Summary of HER2 positive cases. Dark blue, high frequency of HER2 positive cases; white, low frequency of HER2 positive cases.

To clarify the molecular context in which *ERBB2* and *EGFR* gene amplifications and overexpression occur we performed an in-depth study of *EGFR*, *ERBB2*, and *ERBB3* alterations in relation to existing urothelial carcinoma molecular subtypes. In essence, urothelial carcinomas could be divided into two distinct categories based on *EGFR*, *ERBB2*, and *ERBB3* expression and genomic alterations. One category showed high ERBB2/ERBB3 expression with concomitant high frequencies of clinically HER2 positive patients. This category coincided very well with the molecular subtypes Uro and GU (Summarized in Figure 6). The Uro and GU subtypes combined correspond to the “Luminal” subtypes of urothelial carcinoma as described by Choi *et al*. [16], Damrauer *et al*. [17], and the TCGA Clusters I and II [18].

The increased ERBB2 amplification and expression events seen in the Uro subtype seems to be a part of the Uro progression process from non-muscle invasive to muscle invasive growth as the frequency of HER2 positive cases increased from 11% in the non-muscle invasive UroA to 54% in the more advanced and muscle invasive UroC. In contrast ERBB2 amplification and expression seems to be a founding feature of the GU phenotype [10, 19], even though no separate and distinct “HER2 subtype” of UC analogous the breast cancer “HER2 enriched” have emerged. Uro tumors differ from GU tumors by exhibiting high frequencies of *FGFR3* and *PIK3CA* mutations [10] and in muscle invasive urothelial carcinoma *FGFR3* mutations are heavily skewed towards the Uro subtype [19]. Furthermore, bi-allelic loss of the *CDKN2A* locus and overexpression of *CCND1* are frequent events in the Uro subtype, indicating a dependency on mitogenic MAPK/PI3-K signaling for cell proliferation [19]. In contrast, genomic events associated with the GU subtype, *i.e*., *RB1* loss, *TP53* mutation, and *E2F3* gene amplification, allow for a more uncontrolled proliferation [19]. In addition, GU tumors, but not UroA tumors, strongly express FOXM1, a direct downstream target of ERBB2 (HER2) [20], and a FOXM1 related gene signature [13]. Furthermore, in breast cancer FOXM1 and HER2 expression is tightly correlated [21]. Consequently, it may be expected that “clinical HER2 positivity” may have different implications in Uro and GU due to context differences. Additionally, the Urothelial-like subtype is to some extent analogous to the ESR1 expressing breast cancer “Luminal A” subtype [13] that responds less well to HER2 targeted therapy [22, 23]. We also noted that less than half of the ERRB2 amplifications found in tumors of the Basal/SCC-like subtype led to increased mRNA and protein levels, indicating that genomic amplification of 17q12 in Basal/SCC-like, as detected by HER2 ISH analyses, does not have the same consequence as in Uro and GU tumors. The strong association between ERBB2 and ERBB3 overexpression in Uro and GU HER-positive cases, but not in the Basal/SCC-like HER2-positive cases, should also be considered, as ERBB3 has been shown to be essential for ERBB2 driven tumor formation and maintenance [24–26]. Hence, recruiting urothelial carcinoma patients for HER2 directed therapy almost certainly have to take the molecular context *i.e*., molecular subtypes, into consideration.

Even though clinically positive HER2 tumors showed heterogeneity in respect to molecular context, the EGFR positive samples represents a subset of well-defined urothelial carcinoma tumors. EGFR expressing cases coincided well with tumors defined as Basal/SCC-like using the consensus definition *i.e*., KRT5/KRT14 high and FOXA1/GATA3 low [9]. In line with this, urothelial tumors of the Basal/SCC-like subtype respond well to erlotinib in experimental systems [3]. The Basal/SCC-like subtype show extensive molecular similarities with the breast cancer basal-like subtype as well as the SCC subtype of lung carcinomas [13, 27]. Consequently, lessons learned from EGFR targeted treatment in these tumor types may be translated into treatment of Basal/SCC-like urothelial carcinomas. The fact that the EGFR/ERBB2 and ERBB3 expression ratio readily identified two distinct classes of urothelial carcinomas strongly associated with the independently determined molecular subtypes shows that the status of the EGFR, ERBB2, and ERBB3 receptors may represent fundamentally different tumor cell phenotypes (Figure 6).

In conclusion, we argue that future clinical trials using targeted therapies against ERBB2 (HER2) may have to take the molecular context *i.e*., molecular subtypes, into consideration. It may be that clinical trials up to now have not fully appreciated the molecular background, and thus the molecular heterogeneity, in which activated ERBB2 and EGFR signaling systems are operating in. We believe that a proper tumor classification is critical for arriving at thorough conclusions during the evaluation process of new treatment regimes.

## MATERIALS AND METHODS

### Tumor cohort and data availability

The present investigation makes use of two cohorts, one reference cohort containing all tumor stages and one cohort of advanced tumors. The reference cohort consists of 292 cases established from previously published data [10, 12] (GSE32894 and GSE32549) and includes all tumor stages (123 Ta, 88 T1, 79 ≥T2, and 2 Tx) and grades (54 G1, 96 G2, 141 G3, and 1 Gx, WHO1999). Tumors were classified as Urothelial-like A (UroA, *n* = 149), Urothelial-like B (UroB, *n* = 21), Genomically Unstable (GU, *n* = 90), or Squamous Cell Carcinoma-like (SCC-like, *n* = 32). Among the 292 samples, 249 had copy number array data derived from one or more platforms [12, 14, 28, 29]. Tumors showing narrow amplifications peaks on the lower resolution array platforms were hybridized to a custom made high density zoom-array with an average genome-wide probe spacing of 17 500 bp and between 7 000 - 12 000 bp in select target regions [30]. All copy number profiles and copy number calls for *EGFR*, *ERBB2*, and *ERBB3* were manually evaluated. For 217 samples both copy number data and IHC data were available.

The advanced cohort consists of 307 formalin-fixed paraffin-embedded TUR-B samples from patients that underwent radical cystectomy between 2006 and 2011. Of these, 243 were muscle invasive in the pathological TUR-B evaluation. Generation of gene expression and immunohistochemistry data is described in detail by Sjödahl *et al*. 2017 [15]. Briefly, tissue-microarray cores were positioned to yield high tumor cell content and to be morphologically and histologically representative of the tumor. RNA extraction was performed on macro-dissected tissue areas located close to the TMA core sites, using HighPure isolation kits (Roche). RNA was amplified and labeled using SensationPlus kits (Affymetrix) and hybridized to the Gene ST 1.0 platform (Affymetrix). Raw- and normalized data is deposited in Gene Expression Omnibus under GSE83586. The tumors were classified using a combination of global mRNA gene expression analysis and IHC analysis into Urothelial-like, Urothelial-like B, Genomically Unstable, Basal/SCC-like, Mesenchymal Infiltrated, and Small cell/Neuroendocrine subtypes as detailed in Sjödahl *et al*. 2017 [15]. Six heavily infiltrated cases could not be assigned to any of the subtypes. These were categorized as “infiltrated”, and referred to as “Inf” in the text. Array CGH data was not available for this cohort. Informed consent was obtained from all patients and the study was approved by the Local Ethical Committee of Lund University in accordance with the Helsinki declaration.

### Immunohistochemistry

Tissue microarrays (two 1.0 mm cores per tumor, 4 μm sections) were analyzed with antibodies against EGFR (3C6, Ventana), ERBB2 (4B5, Ventana), ERBB3 (Dako, DAK-H3-IC). TMA cores were evaluated as blinded digitalized image files. Each TMA slide contained 120 randomly selected tumor cores to obtain an efficient distribution between low (blank) and high expressing cases. Antibody dilutions were tuned to obtain a staining ranging from blank to intensive staining. IHC stainings for EGFR, ERBB2, and ERBB3 were quantitatively scored from 0 to 3. When hetrogeneous staining was observed within a single core, the fraction of tumor cells with each score was recorded and multiplied with the intensity for a tumor cell score (TCS). The mean tumor cell score of core pairs from the same sample was calculated. Immunohistochemistry analyses of KRT5, KRT14, GATA3, FOXA1, FGFR3, CCND1, CDKN2A (p16), and RB1 was as reported by Sjödahl *et al*. 2017 [15].

### Clinical HER2 status

Clinical HER2 status was assessed using dual *in-situ* hybridization (ISH) and IHC according to Swedish guidelines for HER2 testing in breast cancer [31]. The clinical scoring was performed on a separate section, independently evaluated by an experienced bladder and breast cancer pathologist (GC) using Ventana Pathway HER2 4B5 and Ventana Dual ISH assay HER2 DNP and CHR17 DIG probes on the BenchMark Ultra platform. The IHC score (0, 1+, 2+, 3+) was determined by the highest staining observed in >10% of the tumor cells. An *ERBB2*/CEN17 ISH ratio ≥ 2, or ≥ 4 *ERBB2* copies when CEN17 was not determinable, was used for amplification calls. In samples with two TMA cores the highest ratio was used for calling amplifications. HER2 amplified tumors with IHC 2+ scores in > 10% of cells were considered HER2-positive. The inter-observer agreement between the IHC core intensity scores by GS and the clinical re-evaluation by GC was excellent (kappa 0.807 with equal weights).

## SUPPLEMENTARY FIGURE



## Abbreviations

TMA: Tissue Microarray
IHC: Immunohistochemistry
TCS: Tumor Cell Score
ISH: In-Situ Hybridization
CEN17: Chromosome 17 centromere
