# Supplementary material for: HER2 and EGFR amplification and expression in urothelial carcinoma occurs in distinct biological and molecular contexts
Source: Oncotarget. 2017 Mar 24;8(30):48905–14. doi: 10.18632/oncotarget.16554 (PMC5564734; doi:10.18632/oncotarget.16554)
Supplement: Supplementary file 1 [file oncotarget-08-48905-s001.pdf]

HER2 and EGFR amplification and expression in urothelial carcinoma occurs in distinct biological and molecular contexts

Supplementary Materials

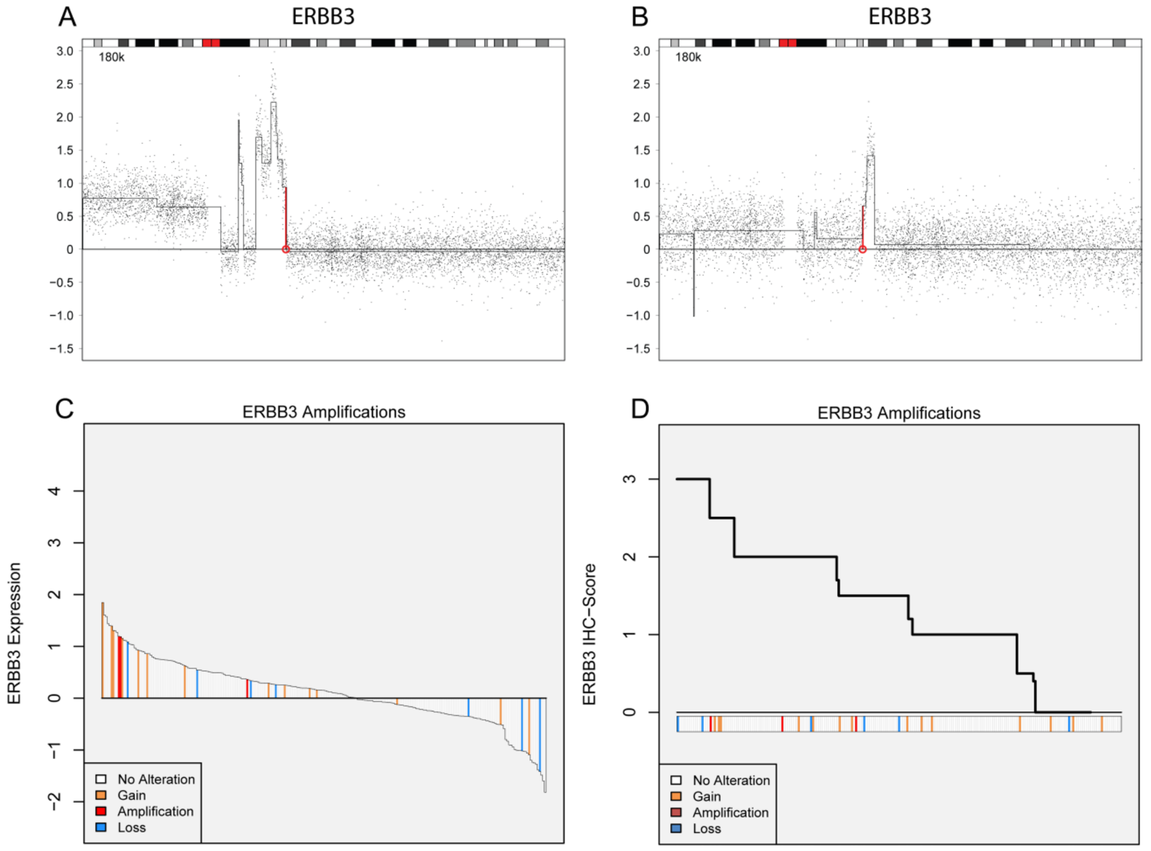

Supplementary Figure 1: ERBB3 copy number alterations, mRNA expression, and protein expression (IHC). (**A**) and (**B**). Examples of focal amplification events spanning *ERBB3* on chromosome 12. The genomic position of *ERBB3* is indicated by a red circle. (**C**) Ranked mRNA gene expression levels for 249 samples with both gene expression and copy number aberration data. (**D**) Ranked ERBB3 immunohistochemistry scores (TSC) for 251 samples with both IHC and copy number aberration data. ERBB3 gene copy number levels; focal amplification, red; gain, orange; loss, blue.
